# Nonlinear Optical Rectification in an Inversion-Symmetry-Broken Molecule near a Metallic Nanoparticle

**DOI:** 10.3390/nano12061020

**Published:** 2022-03-21

**Authors:** Natalia Domenikou, Ioannis Thanopulos, Vassilios Yannopapas, Emmanuel Paspalakis

**Affiliations:** 1 Materials Science Department, School of Natural Sciences, University of Patras, 26504 Patras, Greece; domenikou.n@gmail.com (N.D.); paspalak@upatras.gr (E.P.); 2 Department of Physics, National Technical University of Athens, 15780 Athens, Greece; vyannop@mail.ntua.gr

**Keywords:** NOR, PDMs, asymmetric two-level quantum system, plasmonic nanoparticle, zinc–phalocyanine molecular complex

## Abstract

We study the nonlinear optical rectification of an inversion-symmetry-broken quantum system interacting with an optical field near a metallic nanoparticle, exemplified in a polar zinc–phthalocyanine molecule in proximity to a gold nanosphere. The corresponding nonlinear optical rectification coefficient under external strong field excitation is derived using the steady-state solution of the density matrix equations. We use *ab initio* electronic structure calculations for determining the necessary spectroscopic data of the molecule under study, as well as classical electromagnetic calculations for obtaining the influence of the metallic nanoparticle to the molecular spontaneous decay rates and to the external electric field applied to the molecule. The influence of the metallic nanoparticle to the optical rectification coefficient of the molecule is investigated by varying several parameters of the system, such as the intensity and polarization of the incident field, as well as the distance of the molecule from the nanoparticle, which indirectly affects the molecular pure dephasing rate. We find that the nonlinear optical rectification coefficient can be greatly enhanced for particular incident-field configurations and at optimal distances between the molecule and the metallic nanoparticle.

## 1. Introduction

The manipulation and tuning of the nonlinear optical properties of quantum systems (QSs) is important in various photonic applications, as it can enhance the generally weak interaction between light and quantum matter. A method recently used for the enhancement of nonlinear optical processes in QSs is the placement of the QSs near plasmonic (mainly metallic) nanostructrures. The localized surface plasmons supported by plasmonic nanostructures interact with the excitations of the QSs and enhance the nonlinear optical response of the QSs. Some examples of nonlinear optical phenomena that have been studied in coupled quantum plasmonic nanostructures are nonlinear Fano resonances in the energy absorption [1,2,3,4,5], optical transparency and gain without inversion [6,7,8,9,10], controlled four-wave mixing and Kerr nonlinearity [11,12,13], as well as optical bistability [14,15,16].

The above phenomena have been studied in QS with inversion symmetry. Asymmetric QSs that do not possess inversion symmetry give rise to second-order nonlinearities due to the occurrence of otherwise forbidden electronic transitions. At the same time, the permanent electric dipole moments (PDMs) which exist in asymmetric QSs also interact with light and can therefore modify the nonlinear optical response of the QSs. Some of the nonlinear optical phenomena that have been studied in isolated asymmetric QSs are second-order harmonic generation [17], two-photon phase conjugation [18], enhanced light emission at the terahertz [19,20], creation of high-order harmonic generation [21], saturation of the nonlinear optical response [22], and efficient generation of correlated photon pairs [23]. The modification of the population inversion as well as the nonlinear and quantum optical properties of bichromatically driven asymmetric QSs have also attracted significant attention [24,25,26,27]. Recently, work has been devoted to the influence of the PDMs on the optical response of asymmetric QSs in cavities [28,29]. Second-order nonlinear optical effects have also been studied in QSs near plasmonic nanostructures. For example, plasmon-enhanced second-harmonic generation [30,31,32] and difference-frequency generation [33] have been analyzed in semiconductor quantum dots coupled with metallic nanoparticles (MNPs).

An important second-order nonlinear optical effect is the nonlinear optical rectification (NOR), which has also been studied in molecules and semiconductor quantum dots near plasmonic nanostructrures [32,34,35]. Specifically, Thanopulos et al. [34] studied the enhancement of the NOR coefficient of molecules placed near a periodic plasmonic nanostructure under very weak excitation. The strong enhancement of the NOR coefficient stems from the suppression of the spontaneous decay rate of the molecules due to the Purcell effect induced by the periodic plasmonic nanostructure. Furthermore, Evangelou [32] found either suppression or enhancement for the NOR coefficient of a quantum dot near a spherical MNP under very weak excitation. In that work, the modification of the NOR coefficient was attributed to the Purcell effect. The influence of the light intensity to the NOR coefficient of a semiconductor quantum dot near a MNP, without taking into account the Purcell effect, was studied by Carreño et al. who reported saturation effects [35].

In this work, we study the NOR of an inversion-symmetry-broken QS which is placed near a MNP, both interacting with an incident electromagnetic field. More specifically, we consider a polar zinc–phthalocyanine molecule in the vicinity of a gold nanosphere. First, we derive the corresponding NOR coefficient under external strong field excitation using the steady-state solution of the relevant density matrix equations. Next, we use *ab initio* electronic structure calculations to determine the necessary spectroscopic data of the molecule under study. In addition, we perform numerical electromagnetic calculations in order to (a) calculate the modified, due to the presence of the MNP, molecular spontaneous rates and (b) the electric field acting on the molecule which is the incident field plus the field scattered off the MNP. In order to quantify the effect of the MNP on the NOR coefficient, we vary different parameters of the calculation setup, such as the polarization and intensity of the electric field, as well as the molecular pure dephasing rate. The variation of the dephasing rate is achieved by varying the distance between the molecule and the MNP. Lastly, we provide the optimal values of the above parameters for maximizing the enhancement of NOR coefficient.

The paper is organized as follows. In Section 2, we derive the equation for the NOR coefficient in the presence of the electromagnetic field using the density matrix equations for the QS under study. In Section 3, we first introduce the electronic-structure calculations for the molecule and then present the results for the NOR coefficient in the absence or presence of the MNP for various parameters of the system. Finally, in Section 4, we summarize our findings.

## 2. Theoretical Model

**Figure 1:**
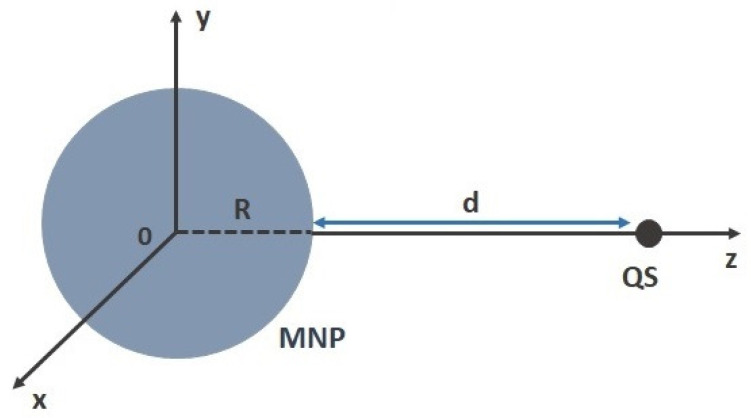
The quantum system (QS) at distance *d* from the surface of the metallic nanoparticle (MNP) with radius *R*.

We consider a hybrid structure composed of a polar two-level QS and a MNP, as depicted in Figure 1, where the distance between the QS and the surface of the MNP is denoted by *d*. The ground (|1〉) and the excited state (|2〉) of the QS have energies $\hbar \omega_{1}$ and $\hbar \omega_{2}$, respectively, while the corresponding transition dipole moment is given by ${\vec{\mu }}_{12}$. The two states of the QS feature unequal PDMs, ${\vec{\mu }}_{11}$ and ${\vec{\mu }}_{22}$, respectively, due to the absence of the inversion symmetry in the QS. As an MNP, we consider a gold (Au) nanoparticle of radius R=80 nm; the local dielectric function of Au is obtained from spectroscopic data [36]. Both the QS and the MNP are embedded in air (refractive index n=1).

The Hamiltonian of the hybrid system is given by

(1) $$H\left(t\right)=\sum_{i=1,2}\left(\hbar \omega_{i}-{\vec{\mu }}_{ii}\cdot \vec{E}\left(t\right)\right)\left|i\rangle \langle i\right|-({\vec{\mu }}_{12}\vec{E}\left(t\right)|1\rangle \langle 2|+H.c.)\;,$$

with $\vec{E}\left(t\right)={\vec{E}}_{0}e^{-i\omega t}+{\vec{E}}_{0}^{*}e^{i\omega t}$, ω being the angular frequency of the field and ${\vec{E}}_{0}$ being the modified external field amplitude due to the presence of the MNP.

In this work, we consider the external electric field being polarized either along the *z*-axis or along the *x*-axis, corresponding to either radially or tangentially polarized external field with respect to the surface of the MNP, respectively (see Figure 1). We also assume that the dipole moments ${\vec{\mu }}_{12}$, ${\vec{\mu }}_{11}$ and ${\vec{\mu }}_{22}$ are always parallel to the interacting field polarization ${\vec{E}}_{0}$. Therefore, in the following, we suppress the polarization indices of the dipole moments and external electric fields, and denote $\mu_{12}=\left|{\vec{\mu }}_{12}\right|$ and $E_{0}^{i}={\vec{E}}_{0}$(i=x,z).

The amplitude of the external electric field, $E_{0}^{i}$, at the position of the molecule is related to the amplitude of incident field, $E_{0f}$, by the modified field factor $|E_{i}\left|\equiv \right|E_{0}^{i}\left|/\right|E_{0f}|$, which is obtained by the Mie scattering method [37]. We note that $E_{0f}$ is related to the incident irradiation intensity *I* by $|E_{0f}{|}^{2}=2I/nc\epsilon_{0}$.

**Figure 2:**
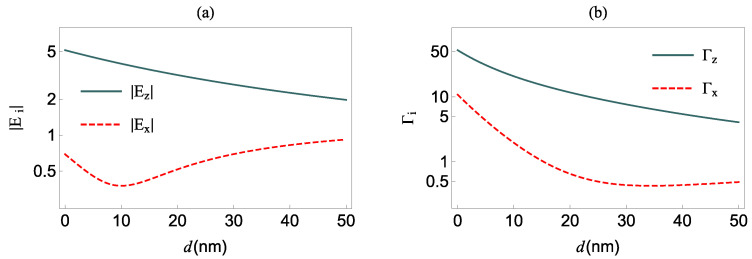
(**a**) The modified field factors of the field applied to the molecule and (**b**) the Purcell enhancement factors of the QS as function of the distance d of the QS to the surface of the MNP.

The corresponding modified field factors are shown in panel (a) in Figure 2 as functions of the distance *d* between the QS and the surface of the MNP, for QS energy 1.99 eV. The $|E_{z}|$ factor decreases monotonically with increasing *d*, while the $|E_{x}|$ factor initially decreases below 1, assumes the minimum value 0.1 at d≈10 nm, and then starts to increase monotonically. In panel (b) of Figure 2, we present the Purcell enhancement factors $\Gamma_{i}=\Gamma_{0}^{i}/\Gamma_{0f}^{i}$, (i=x,z), of a QS with transition dipole moment oriented along the *i*-axis, and free-space decay rate value of $\Gamma_{0f}^{i}=\omega_{21}^{3}\mu_{12}^{2}/3\pi c^{3}\hbar \epsilon_{0}$, with $\omega_{21}=\omega_{2}-\omega_{1}$. These are obtained from the electromagnetic Green’s tensor [37]. We observe that the $\Gamma_{z}$ enhancement factor decreases monotonically, and, in general, it is larger than the $\Gamma_{x}$ enhancement factor. The latter decreases up to d≈34.5 nm and increases thereafter very slowly.

In order to describe the dynamics of the system within the density matrix approach, we define the population difference $\Delta \left(t\right)=\rho_{22}\left(t\right)-\rho_{11}\left(t\right)$ and the optical coherence σ(t)

(2) $$\sigma \left(t\right)=\rho_{12}\left(t\right)e^{-i\omega t+\frac{2i|\mu |}{\hbar \omega }\left(E_{R}\sin\left(\omega t\right)-E_{I}\cos\left(\omega t\right)\right)},$$

where $\mu =\mu_{22}-\mu_{11}$. In Equation (2), the modified field amplitude is written as a complex number $E_{0}^{i}=E_{R}+iE_{I}$. It follows that the equations for optical coherence and population difference be given by

(3) $$\begin{array}{cc} \dot{\sigma }\left(t\right)= & -\left[\frac{1}{T_{2}}+i\delta \right]\sigma \left(t\right)\\ \; & +[i\Omega_{R}\left(A+B\right)+\Omega_{I}\left(A-B\right)]\Delta \left(t\right), \end{array}$$

(4) $$\begin{array}{cc} \dot{\Delta }\left(t\right)= & -\frac{1}{T_{1}}\left[\Delta \left(t\right)+1\right]\\ \; & -2\left[i\Omega_{R}\left(A+B\right)+\Omega_{I}\left(A-B\right)\right]\sigma^{*}\left(t\right)\\ \; & +2\left[i\Omega_{R}\left(A^{*}+B^{*}\right)-\Omega_{I}\left(A^{*}-B^{*}\right)\right]\sigma \left(t\right), \end{array}$$

with $\delta =\omega -\omega_{21}$ being the field detuning, and $\Omega_{m}=\mu_{12}E_{m}/\hbar$ (m=R,I) being the real and imaginary part of the complex Rabi frequency expressed as $\Omega_{0}=\Omega_{R}+i\Omega_{I}=\mu_{12}E_{0}^{i}/\hbar$. In Equations (3) and (4), we also introduced

(5a) $$\begin{array}{cc} \; & A=\sum_{n=-\infty }^{+\infty }{\left(-i\right)}^{n}J_{n}\left(\alpha_{R}\right)J_{n}\left(\alpha_{I}\right), \end{array}$$

(5b) $$\begin{array}{cc} \; & B=\sum_{n=-\infty }^{+\infty }{\left(-i\right)}^{n}J_{n+2}\left(\alpha_{R}\right)J_{n}\left(\alpha_{I}\right), \end{array}$$

with $J_{n}\left(\cdot \right)$ denoting the *n*th-order ordinary Bessel function, and $\alpha_{m}=\frac{2|\mu |E_{m}}{\hbar \omega }$ (m=R,I). We note that for a nonpolar system, i.e., μ=0, the parameters *A* and *B* are equal to 1 and 0, respectively [35].

We also note that $T_{1}$ and $T_{2}$ in Equations (3) and (4) are influenced by the presence of the MNP, due to the relation $\frac{1}{T_{2}}=\frac{1}{2T_{1}}+\gamma_{d}$, where $T_{1}=1/\Gamma_{0}^{i}$, (i=x,z), is the directional relaxation time, $T_{2}$ is the corresponding dephasing relaxation time, and $\gamma_{d}$ is the pure dephasing rate of the QS, respectively.

The steady-state solutions of Equations (3) and (4) are obtained as

(6) $$\begin{array}{cc} \sigma_{\infty }=-T_{2}[i\Omega_{R} & \left(A+B\right)+\Omega_{I}\left(A-B\right)]\\ \; & \times \frac{1-iT_{2}\delta }{1+T_{2}^{2}\delta^{2}+4T_{1}T_{2}{\left|\Omega_{0}^{*}A+\Omega_{0}B\right|}^{2}}, \end{array}$$

(7) $$\Delta_{\infty }=-\frac{1+T_{2}^{2}\delta^{2}}{1+T_{2}^{2}\delta^{2}+4T_{1}T_{2}{\left|\Omega_{0}^{*}A+\Omega_{0}B\right|}^{2}}.$$

For the calculation of the NOR coefficient, we need the induced polarization *P* of the QS, which is given by $P=P_{1}+P_{2}$, with

(8) $$P_{1}=\frac{1}{2}N\mu \left(1+\Delta \right),$$

(9) $$P_{2}=N\mu_{12}\left(\rho_{12}+\rho_{21}\right).$$

where *N* is the effective electron volume density of the QS. The first term, $P_{1}$, which includes the PDMs, is the main contribution to *P*, while the second term, including the transition dipole $\mu_{12}$, is a small contribution to *P*, which, of course, becomes important when the PDMs are equal [22].

The NOR coefficient can be determined using the Equations (7) and (8), in conjunction with the equation $P=\epsilon_{0}\chi_{0}^{\left(2\right)}{\left|E_{0}^{i}\right|}^{2}/4$, due to the following identity [35]:

(10) $$\frac{\epsilon_{0}\chi_{0}^{\left(2\right)}{\left|E_{0}^{i}\right|}^{2}}{4}=N\mu_{12}\left(\sigma_{\infty }B+\sigma_{\infty }^{*}\mu \right)+\frac{N}{2}\mu \left(1+\Delta_{\infty }\right)\;,$$

which finally leads in our case to the $\chi_{0}^{\left(2\right)}$ coefficient, which is central in this work, given by

(11) $$\begin{array}{cc} \chi_{0}^{\left(2\right)}\left(\delta =0,I\right)= & \frac{36N\pi^{2}c^{6}\epsilon_{0}\left|\mu \right|}{\omega_{21}^{2}\left(\Gamma_{i}^{2}+2\Gamma_{i}{\bar{\gamma }}_{d}\right)\mu_{12}^{2}}\\ \; & \times \frac{1}{1+\frac{36\pi^{2}c^{6}\hbar^{2}\epsilon_{0}^{2}\delta^{2}}{\omega_{21}^{2}{\left(\Gamma_{i}+2{\bar{\gamma }}_{d}\right)}^{2}\mu_{12}^{4}}+\frac{144\pi^{2}c^{5}\epsilon_{0}{\left|E_{i}\right|}^{2}I}{\omega_{21}^{6}n\left(\Gamma_{i}^{2}+2\Gamma_{i}{\bar{\gamma }}_{d}\right)\mu_{12}^{2}}}\;, \end{array}$$

with $\bar{\gamma_{d}}=\gamma_{d}/\Gamma_{0f}^{i}$ (i=x,z). We note that the values of $\Gamma_{i}$ and $|E_{i}|$ depend on the distance *d* between the QS and the MNP. We also note that in deriving Equation (11), we have introduced the approximation A=1 and B=0 in Equations (6), (7), and (10), since the numerical values of these two parameters are very close to 1 and 0, respectively. In the next section, we present the results obtained by applying Equation (11) on a realistic molecular QS.

## 3. Results and Discussion

### 3.1. The Zinc–Phthalocyanine Molecular Complex

**Figure 3:**
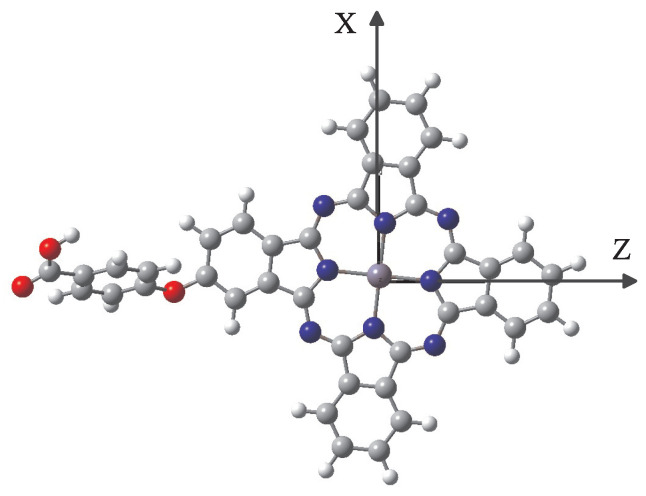
The polar molecular complex used: the zinc–phthalocyanine complex is composed of carbon (gray), hydrogen (white), oxygen (red), nitrogen (blue), and zinc (light blue) atoms. The complex is not planar; however, the phthalocyanine part of the complex is planar, coinciding with the zx-plane, as schematically shown.

We demonstrate the nonlinear optical response of a polar two-level QS, as presented in the previous section on a zinc–phthalocyanine molecular complex shown in Figure 3, which has recently been synthesized [38]. We chose this molecule because its large transition dipole moments between the ground and first excited electronic state, as well as the notable difference between the corresponding PDMs due to its inversion symmetry violation, lead to pronounced NOR effects. We stress that this is just a typical molecule that has these properties, and the effects that we describe below also apply to other molecules that possess similar properties. In fact, currently there is intensive interest in the nonlinear optical properties of molecules under the interaction with laser fields and several interesting and potentially useful experimental results have been presented, see, e.g., the works in [39,40].

In our calculations presented below, the ground and first singlet excited electronic states of this complex are the states |1〉 and |2〉 of the QS, respectively. The corresponding molecular spectroscopic parameters are obtained by *ab initio* electronic structure methods, after geometry optimization of the molecular structure of state |1〉 at the DFT/B3LYP/6-311$+G^{*}$ and for state |2〉 at the TD-DFT/B3LYP/6-31-$G^{*}$ level of theory [41].

From the *ab initio* calculations, we obtain the QS transition energy $\hbar \omega_{21}=1.99$ eV, as well as the values of the PDMs and the transition dipole moments, as given in Table 1. Moreover, the free-space spontaneous decay widths used are $\Gamma_{0f}^{z}\approx 13.6$ MHz and $\Gamma_{0f}^{x}\approx 22.3$ kHz when the corresponding transition dipole moment is along the *z*-axis and the *x*-axis, respectively.

### 3.2. NOR of the Zinc–Phthalocyanine Complex

**Figure 4:**
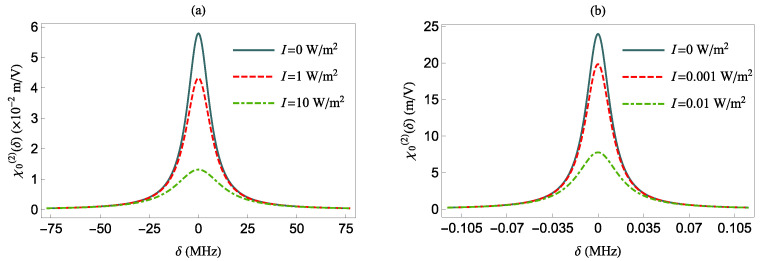
The $\chi_{0}^{\left(2\right)}$ as function of δ for various intensities in absence of the MNP and $\gamma_{d}=0$. The QS transition dipole moment is along the *z*-axis (**a**) and along the *x*-axis (**b**).

**Figure 5:**
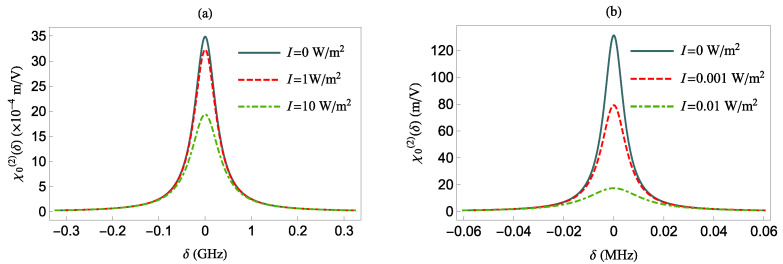
The $\chi_{0}^{\left(2\right)}\left(\delta \right)$ as function of δ for various intensities in the presence of the MNP and $\gamma_{d}=0$. (**a**) The $\mu_{12}$ is along the *z*-axis and d=50 nm. (**b**) The $\mu_{12}$ is along the *x*-axis and d=34.5 nm.

In Figure 4 and Figure 5, we present the NOR coefficient $\chi_{0}^{\left(2\right)}$ in the absence or presence of the MNP as a function of various parameters in the case of no pure dephasing, $\gamma_{d}=0$.

More specifically, in Figure 4, we present the $\chi_{0}^{\left(2\right)}\left(\delta \right)$ at various intensities *I*, for a QS with $\gamma_{d}=0$ and a transition dipole oriented either along the *z*-axis [panel (a)] or along the *x*-axis [panel (b)], in the absence of the MNP (i.e., d→∞). The latter means that ${\bar{\gamma }}_{d}=0$, $\Gamma_{i}=1$, and $|E_{i}|=1$. The largest values of $\chi_{0}^{\left(2\right)}\left(\delta \right)$, for all intensities *I*, are obtained for δ=0. The corresponding values for $\chi_{0}^{\left(2\right)}\left(\delta =0\right)$ are given by

(12) $$\chi_{0}^{\left(2\right)}\left(\delta =0,I=0;d=\infty \right)=\frac{36N\pi^{2}c^{6}\epsilon_{0}\left|\mu \right|}{\omega_{21}^{2}\mu_{12}^{2}}.$$

and

(13) $$\chi_{0}^{\left(2\right)}\left(\delta =0,I\neq 0;d=\infty \right)=\frac{\chi_{0}^{\left(2\right)}\left(\delta =0,I=0;d=\infty \right)}{1+\frac{144\pi^{2}c^{5}\epsilon_{0}I}{\omega_{21}^{6}n\mu_{12}^{2}}}\;.$$

We observe that the $\chi_{0}^{\left(2\right)}\left(\delta =0\right)$ value in each panel is maximized in the absence of the electric field. When I≠0, the system reaches saturation as the field intensity increases, resulting in a smaller value for $\chi_{0}^{\left(2\right)}\left(\delta =0\right)$ due to the larger denominator in Equation (13). Moreover, since $\chi_{0}^{\left(2\right)}\left(\delta =0\right)$ is inversely proportional to $\mu_{12}^{2}$, the NOR coefficient assumes larger values in the bottom panel than in the top panel of Figure 4. This is because $\mu_{12}^{2}$ is smaller along the *x* direction than in the *z* direction, as shown in Table 1.

In order to assess the influence of the MNP on the NOR coefficient, in Figure 5, we investigate the NOR coefficient at various intensities *I*, for $\gamma_{d}=0$, in the presence of the MNP, i.e., for $\Gamma_{i}\neq 1$ and $|E_{i}|\neq 1$. The QS is located at d=50 nm and d=34.5 nm while its transition dipole moment is along the *z*- [panel (a)] and *x*-axis [panel (b)], respectively. These distance values are chosen in order to have the smallest Purcell enhancement factor in each case, according to Figure 2. Again here, the largest values of $\chi_{0}^{\left(2\right)}$ for all values of *I* are obtained at δ=0 and they are provided by

(14) $$\chi_{0}^{\left(2\right)}\left(\delta =0,I=0;d\right)=\frac{\chi_{0}^{\left(2\right)}\left(\delta =0,I=0;d=\infty \right)}{\Gamma_{i}^{2}}\;,$$

and

(15) $$\chi_{0}^{\left(2\right)}\left(\delta =0,I\neq 0;d\right)=\frac{\chi_{0}^{\left(2\right)}\left(\delta =0,I=0;d\right)}{1+\frac{|E_{i}{|}^{2}}{\Gamma_{i}^{2}}\frac{144\pi^{2}c^{5}\epsilon_{0}I}{\omega_{21}^{6}n\mu_{12}^{2}}}\;.$$

In Figure 5, we observe that $\chi_{0}^{\left(2\right)}$ (NOR) is much stronger for tangential polarization of the external field (panel (a)) than for the radial one. By comparing with the results of Figure 4 (absence of MNP), when I=0, $\chi_{0}^{\left(2\right)}$ is suppressed by a factor equal to 0.06 for radial polarization, and enhanced by a factor equal to 5.47 for tangential polarization of the external field. This observation can be rationalized with the help of Equation (14) where it can be seen that the $\chi_{0}^{\left(2\right)}\left(\delta =0,I=0\right)$ is inversely proportional to the $\Gamma_{i}^{2}$ factor in this case. Moreover, when I≠0, the NOR suppression or enhancement, related to the radial and tangential polarization of the external field, is not as pronounced as in the cases shown in Figure 4. This is due to the presence $|E_{i}{|}^{2}/\Gamma_{i}^{2}$ factor in the denominator of Equation (15).

**Figure 6:**
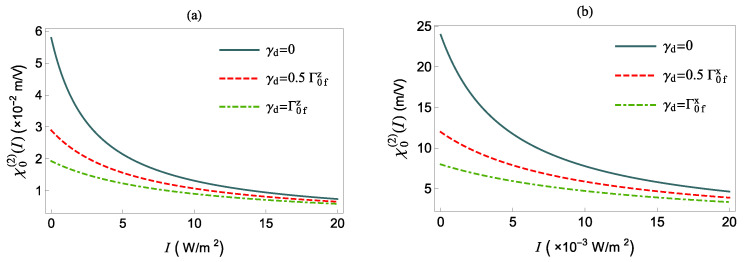
$\chi_{0}^{\left(2\right)}\left(\delta =0\right)$ as a function of intensity *I*, in the absence of the MNP and for various values of $\gamma_{d}$. The QS transition dipole moment is along the *z*-axis (**a**) and *x*-axis (**b**), respectively.

**Figure 7:**
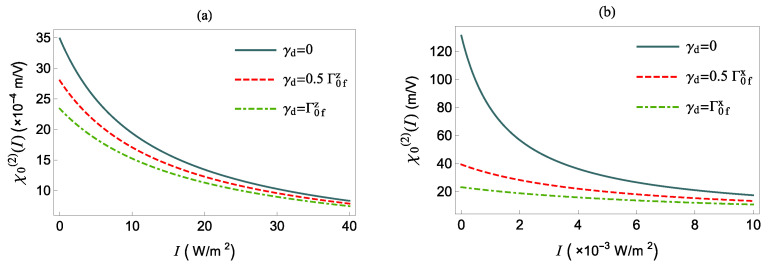
$\chi_{0}^{\left(2\right)}\left(\delta =0\right)$ as a function of the intensity *I* in presence of the MNP for various $\gamma_{d}$. (**a**) The $\mu_{12}$ is along the *z*-axis and d=50 nm. (**b**) The $\mu_{12}$ is along the *x*-axis and d=34.5 nm.

In the next figures, Figure 6 and Figure 7, we study how the pure dephasing rate $\gamma_{d}$ affects the NOR coefficient of the polar QS in the absence or presence of the MNP, respectively.

In Figure 6, we present $\chi_{0}^{\left(2\right)}\left(\delta =0\right)$ as a function of the intensity *I*, for various $\gamma_{d}$, in the absence of the MNP, i.e., for $\Gamma_{i}=1$ and $|E_{i}|=1$. We find that $\chi_{0}^{\left(2\right)}\left(\delta =0\right)$ decreases monotonically as *I* increases due to saturation effects. We note that the field is polarized along the *x*-axis, the saturation requires intensity three orders of magnitude smaller than for the field being polarized along the *z*-axis. For $\gamma_{d}=0$ and no external field, the $\chi_{0}^{\left(2\right)}\left(\delta =0\right)$ coefficient assumes its largest values, according to Equation (13). For non-zero $\gamma_{d}$, the $\chi_{0}^{\left(2\right)}\left(\delta =0\right)$ values in the absence of the MNP are given by

(16) $$\chi_{0}^{\left(2\right)}\left(\delta =0,I;d=\infty \right)=\frac{36N\pi^{2}c^{6}\epsilon_{0}\left|\mu \right|}{\omega_{21}^{2}\left(1+2{\bar{\gamma }}_{d}\right)\mu_{12}^{2}}\cdot \frac{1}{1+\frac{144\pi^{2}c^{5}\epsilon_{0}I}{\omega_{21}^{6}n\left(1+2{\bar{\gamma }}_{d}\right)\mu_{12}^{2}}}\;;$$

accordingly, the nonlinear optical behavior of QS is suppressed due to the presence of the ${\bar{\gamma }}_{d}$ factor in both factors of the product of Equation (16).

Now, in presence of the MNP, for any intensity, the $\chi_{0}^{\left(2\right)}\left(\delta =0\right)$ coefficient for $\gamma_{d}=0$ is given by Equation (15), while in the case of $\gamma_{d}\neq 0$, it is given by

(17) $$\chi_{0}^{\left(2\right)}\left(\delta =0,I;d\right)=\frac{36N\pi^{2}c^{6}\epsilon_{0}\left|\mu \right|}{\omega_{21}^{2}\left(\Gamma_{i}^{2}+2\Gamma_{i}{\bar{\gamma }}_{d}\right)\mu_{12}^{2}}\cdot \frac{1}{1+\frac{144\pi^{2}c^{5}\epsilon_{0}{\left|E_{i}\right|}^{2}I}{\omega_{21}^{6}n\left(\Gamma_{i}^{2}+2\Gamma_{i}{\bar{\gamma }}_{d}\right)\mu_{12}^{2}}}.$$

We note that in Equation (17), for a given non-zero value of $\gamma_{d}$ and *I*, it is the quantities $|E_{i}{|}^{2}$, $\Gamma_{i}^{2}$ and ${\bar{\gamma }}_{d}$ that determine the suppression of the $\chi_{0}^{\left(2\right)}\left(\delta =0\right)$, in comparison with the $\gamma_{d}=0$ cases. Accordingly, in the presence of the MNP, $\chi_{0}^{\left(2\right)}\left(\delta =0\right)$ in Figure 7 is suppressed (for radially polarized field (panel (a))), at twice the intensity, while it is enhanced (tangentially polarized (panel (b) field)), at half of the intensity, when we compare it with the results shown in Figure 6.

**Figure 8:**
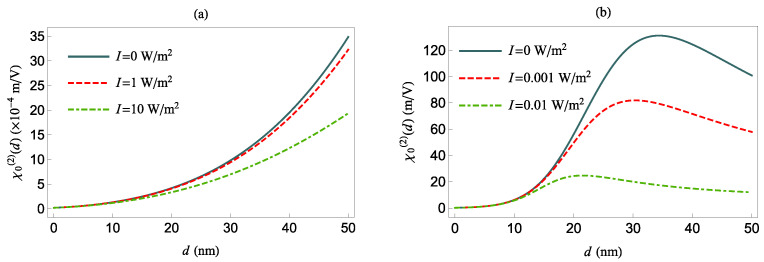
$\chi_{0}^{\left(2\right)}\left(\delta =0\right)$ as a function of *d*, for δ=0 and $\gamma_{d}=0$, and different values of the intensity *I*. The QS transition dipole moment is along the *z*-axis (**a**) and *x*-axis (**b**), respectively.

Next, in Figure 8, we investigate $\chi_{0}^{\left(2\right)}\left(\delta =0\right)$ as a function of the distance *d* between the QS and the MNP, for *z*- (panel (a)) and *x*-polarized (panel (b)) external field, for various values of *I* and no pure dephasing. The values of $\chi_{0}^{\left(2\right)}\left(\delta =0\right)$ for I=0 are given by Equation (14), while for I≠0, they are given by Equation (15).

In the top panel of Figure 8, we observe that $\chi_{0}^{\left(2\right)}\left(\delta =0\right)$ increases with increasing *d*, due to the decrease of $\Gamma_{z}$ for a *z*-oriented transition dipole moment of the QS (see Figure 2b). On the other hand, in the bottom panel of Figure 8, for *x*-oriented external field, we observe that $\chi_{0}^{\left(2\right)}\left(\delta =0\right)$ increases up to d<34.5 nm and decreases monotonically beyond this value. For I≠0, $\chi_{0}^{\left(2\right)}\left(\delta =0\right)$ is suppressed in comparison with the I=0 case due to saturation effects which are evident in both panels in Figure 8. Moreover, we observe that for the tangentially polarized field, the largest value of $\chi_{0}^{\left(2\right)}\left(\delta =0\right)$ occurs at a shorter distance *d* than for the case of the radially polarized field.

**Figure 9:**
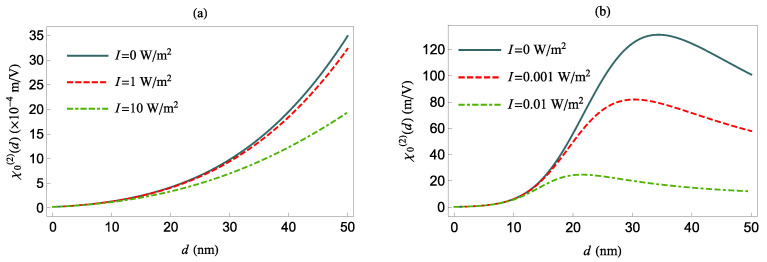
$\chi_{0}^{\left(2\right)}\left(\delta =0\right)$ as a function of *d*, for δ=0 and $\gamma_{d}=0$, and different values of the intensity *I*, but for $\gamma_{d}=\Gamma_{0f}^{i}$. The QS transition dipole moment is along the *z*-axis (**a**) and *x*-axis (**b**), respectively.

Lastly, in Figure 9, we present the nonlinear optical response of the QS as a function of *d* for various *I* and for $\gamma_{d}=\Gamma_{0f}^{i}$. For I=0, $\chi_{0}^{\left(2\right)}\left(\delta =0\right)$ is provided by

(18) $$\chi_{0}^{\left(2\right)}\left(\delta =0;d\right)=\frac{36N\pi^{2}c^{6}\epsilon_{0}\left|\mu \right|}{\omega_{21}^{2}\left(\Gamma_{i}^{2}+2\Gamma_{i}{\bar{\gamma }}_{d}\right)\mu_{12}^{2}}.$$

Accordingly, we observe that $\chi_{0}^{\left(2\right)}\left(\delta =0\right)$ is suppressed by a factor of 1.49 and 5.68 for radially (panel (a)) and tangentially (panel (b)) polarized fields, respectively, when compared with the case of no pure dephasing presented in Figure 9.

For non-zero intensity, in the case of a tangentially polarized field, we observe that as the intensity increases, the highest value of $\chi_{0}^{\left(2\right)}\left(\delta =0\right)$ occurs at slightly larger *d*, than in the case of no pure dephasing (Figure 8b). We note that for $\gamma_{d}>\Gamma_{0f}^{x}$, we find (not shown here) that the highest value of $\chi_{0}^{\left(2\right)}\left(\delta =0\right)$ occurs at about d=34.5 nm; however, in this case, the NOR process becomes very weak, with an almost vanishing $\chi_{0}^{\left(2\right)}\left(\delta =0\right)$.

## 4. Conclusions

We have investigated the influence of an MNP on the phenomenon of NOR in an inversion-symmetry-broken molecular complex, modeled as a polar two-level QS, under external light illumination. Thus, for such a system, we have derived analytically the equations of the NOR coefficient of the QS and obtained the spectroscopic parameters of the molecular complex, a polar zinc–phthalocyanine complex, via *ab initio* methods. The NOR process of an inversion-symmetry-broken QS, under weak field intensity, was found to be proportional to the difference of the PDMs and inversely proportional to the square of the transition dipole moment of the QS.

In particular, we investigated the NOR coefficient as a function of various parameters of the QS, MNP, and incident light configuration, such as the intensity and polarization of the external field, the distance of the QS to the MNP, the directional decay time of the QS, and the pure dephasing rate of the QS.

We found that in the presence of a MNP, when the electric field is polarized radially with respect to the MNP surface, the NOR is suppressed, while it is enhanced when the field is polarized tangentially. For both polarization directions, as the external field intensity increases, the NOR coefficient decreases due to saturation effects. Further, when we increase the pure dephasing rate of the QS, the NOR decreases. We also observed that, for increasing distance *d*, the NOR of the QS is enhanced for radial polarization; in contrast, when the polarization is tangential, NOR is enhanced up to some distance, but then it is slowly suppressed with increasing *d*. Lastly, we found that in presence of the MNP, for the tangential polarization of the field, the NOR process of the QS is more efficient when compared with the free-space case, for certain values of the above variables.

Our findings can be of particular interest for topical quantum technology and nanophotonic applications.

## Figures and Tables

**Table 1:** *Ab initio* obtained values of dipole moments (in Debye) of the ground and first singlet excited electronic state of the molecular complex shown in Figure 3.

Dipole Moments/D	*z*-axis	*x*-axis
$\mu_{11}$	7.2746	1.9557
$\mu_{22}$	6.7071	1.5723
$\mu_{12}$	−3.2476	0.1312

## Data Availability

The data presented in this study are available upon reasonable request from the corresponding author.

## Abbreviations

The following abbreviations are used in this manuscript:

QS	Quantum system
MNP	Metallic nanoparticle
PDMs	Permanent electric dipole moments
NOR	Nonlinear optical rectification
